# Dynamic Scaling in the Growth of a Non-Branching Plant, *Cardiocrinum cordatum*

**DOI:** 10.1371/journal.pone.0045317

**Published:** 2012-09-19

**Authors:** Kohei Koyama, Yoshiki Hidaka, Masayuki Ushio

**Affiliations:** 1 Department of Environmental Science, Ishikawa Prefectural University, Nonoichi, Ishikawa, Japan; 2 Center for Ecological Research, Kyoto University, Otsu, Shiga, Japan; 3 Department of Applied Quantum Physics and Nuclear Engineering, Graduate School of Engineering, Kyushu University, Fukuoka, Japan; University of Zurich, Switzerland

## Abstract

We investigated whole-plant leaf area in relation to ontogenetic variation in leaf-size for a forest perennial herb, *Cardiocrinum cordatum*. The 200-fold ontogenetic variability in *C. cordatum* leaf area followed a power-law dependence on total leaf number, a measure of developmental stage. When we normalized for plant size, the function describing the size of single leaves along the stem was similar among different-sized plants, implying that the different-sized canopies observed at different times in the growth trajectory were fundamentally similar to each other. We conclude that the growth trajectory of a population of *C. cordatum* plant leaves obeyed a dynamic scaling law, the first reported for a growth trajectory at the whole-plant level.

## Introduction

Whole-plant leaf area is a major determinant of plant and ecosystem productivity [1]–[3], and use of allometric relationships to predict whole-plant leaf area is a central topic in plant ecology [4]–[10]. However, one difficulty in modeling whole-plant leaf area is that individual leaves show considerable morphological plasticity in their size. Leaf size is determined by both developmental stage [11]–[15], and by position within a canopy [11]–[13]. To date, this leaf size variation has not been incorporated into whole-plant allometric scaling models [8], [16]; current scaling models are based on the assumption that whole-plant leaf area is proportional to leaf number [6], [7], [16]. The omission of leaf size variation may help to explain why current whole-plant scaling theories do not reliably predict whole-plant leaf area (c.f. [10]). It has been suggested that the metabolic theories for plants will be further improved by incorporating leaf size variation [16]. In this paper, we adopt the concept of dynamic scaling in an attempt to establish such a cross-scale linkage.

Dynamic scaling refers to a phenomenon in which observations of a growing system at different times are similar to each other [17]. Galeano et al. [18] analyzed dynamic scaling of the growth of plant calli (i.e., cultured plant cells). However, to the best of our knowledge, there have been no studies that have reported dynamic scaling at the whole-plant level. We hypothesized that geometric similarity through development would also be expected at the whole-plant level, to maintain overall plant structure to conserve the efficiency of the light-capturing foliar array and mechanical stability during ontogeny. In the present context, we investigated the possibility that one pattern (e.g., leaf size as a function of position within a plant, described by an unknown function), when observed at a particular developmental stage, has the same form when observed at another developmental stage.

One commonly observed correlate of dynamic scaling is that the size of the unit pattern [17], [19] and the size of the entire pattern [18] typically follow a power-law dependence on time. Hence, we investigated the possibility that whole-plant leaf area (the size of the pattern) can be expressed as a power function of total leaf number (a normalized time across developmental stages). Total leaf number across developmental stages is essentially equivalent to the plastochron index or plastochron age, which has been widely applied in studies of the foliar development of herbs [11], [20].

To assess this possibility, we investigated the growth trajectory of the foliar array in a perennial, rosette-forming forest herb, *Cardiocrinum cordatum*. Specifically, we tested the following two hypotheses: (1) the within-plant distribution of leaf size observed at a particular time is described by the same function as that observed at another time (*dynamic scaling*); (2) whole-plant leaf area and mean individual leaf area are power functions of total leaf number across developmental stages (*power law*).

**Figure 1:**
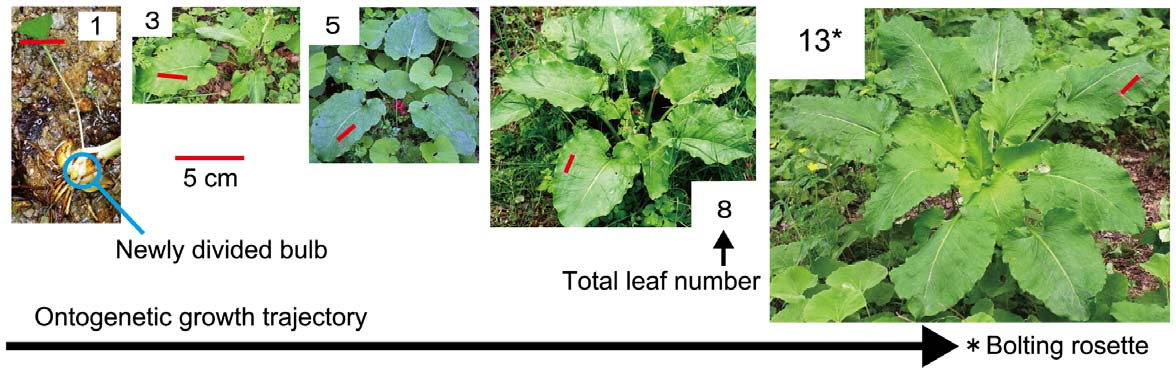
Ontogenetic growth trajectory of *C*. *cordatum* rosettes. The photographs show representative *C*. *cordatum* plants on 10 May 2010. Red scale bars superimposed on each photograph indicate a distance of 5 cm on each leaf. Leaf punch holes and ink markings used for other experiments appear on some of the leaves (photographs by K. Koyama, taken on 10 May 2010).

## Materials and Methods

### Species and Site

*Cardiocrinum cordatum* (Liliaceae) is a monocarpic perennial herb [21]–[23] found in the forest understory. In this species, the entire leaf population dies before the onset of winter, and in the following spring, a new rosette forms with a larger leaf population. When a *C. cordatum* plant flowers, it sets seed and senesces, leaving small bulblets that are produced prior to bolting as asexual propagules [21]–[23]. We sampled a population of *C*. *cordatum* (Fig. 1) from a temperate forest dominated by Japanese alder (*Alnus japonica*, age: 50–60 yr; canopy height: 20 m [24]), located at the Ishikawa Prefectural Forest Experiment Station (36°25′N, 136°38′E; elevation: 220 m), Japan. Mean annual temperature and precipitation at the study site are 13.0°C and 2438 mm, respectively (2003–2007). A more detailed site description has been given elsewhere [25].

**Figure 2:**
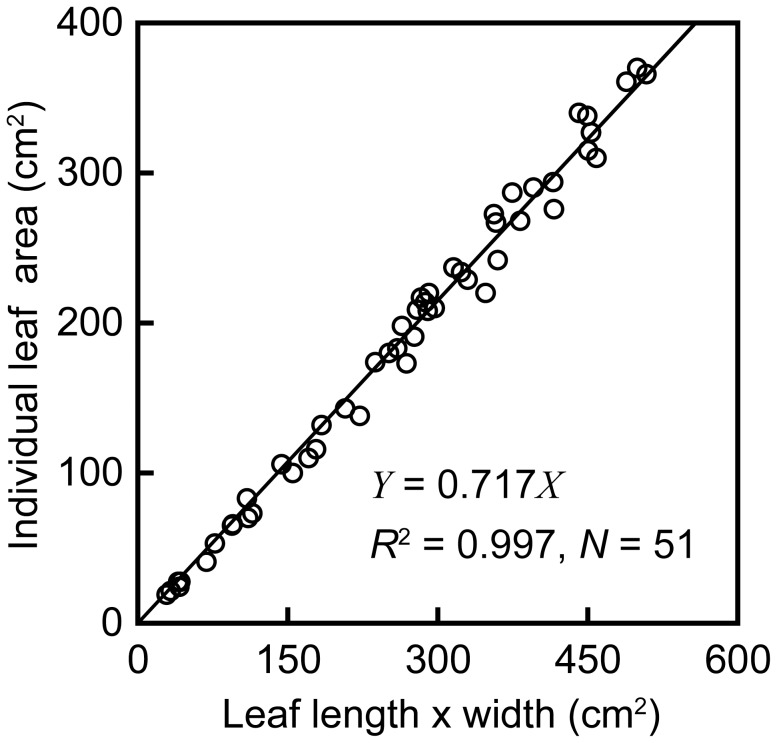
Similarity among individual leaves. Each open circle indicates one harvested leaf (*n* = 51). The solid line shows the ordinary least-squares regression, which was forced through the origin: Leaf area (cm^2^) = 0.717 (length × width) (*r* ^2^ = 0.997, *n* = 51, *p*<0.01).

#### Leaf measurements

A total of 208 leaves on 29 rosettes (1–18 leaves per plant) of *C. cordatum* were sampled on 10 May 2010 (Fig. 1). The smallest plant with only a single leaf (leaf length  = 8.8 cm, leaf area  = 27.6 cm^2^) grew from a bulblet attached to another rosette (Fig. 1). The rosette of the largest plant was >0.8 m in diameter. The five largest rosettes (12–18 leaves) were bolting (i.e., flowering later in the summer); they did not regenerate the following year. Hence, at this study site, we investigated the entire ontogenetic size range of *C. cordatum*. Sampling included fully and partially expanded leaves. Undeveloped leaves, in which laminae were not separate from the stem, were not counted. For smaller, non-flowering rosettes, all leaves had appeared prior to the sampling period. For larger, bolting rosettes, most leaves had appeared prior to the study period, although some additional leaves appeared after sampling as the flowering stem elongated. We measured leaf length (the length of the central vein on each lamina, excluding the petiole) and width (maximum lamina width perpendicular to the central vein) for each leaf. The absolute leaf position was defined by counting the order of appearance from the base of the rosette. Eight of the sampled plants were harvested, and 21 were left at the site for future study. Leaf area was defined as laminar area of one side of each leaf. Laminar area of 51 leaves from harvested plants was measured using a leaf area meter (Li-1300; LI-COR, Lincoln, USA). Individual leaf area is proportional to the product of leaf lamina length and width (Fig. 2). This indicates that individual leaf forms are similar, although the ratio of length to width may vary within an individual plant and population, as has been reported for other species [26], [27]. We fitted an ordinary least squares (OLS) regression of width × length of leaf laminae, using SMATR [28]: Leaf area (cm^2^) = 0.717 (width × length) (*r* ^2^ = 0.997, *p*<0.01, *n* = 51) (Fig. 2). Using this relationship, we calculated leaf area for plants that were not harvested. When the regression line was not forced through the origin, the slope and *r* ^2^ values changed little (slope = 0.735, *r* ^2^ = 0.989, respectively); the 95% confidence intervals of the intercept included the origin, thus the intercept of the regression line was set to zero.

**Figure 3:**
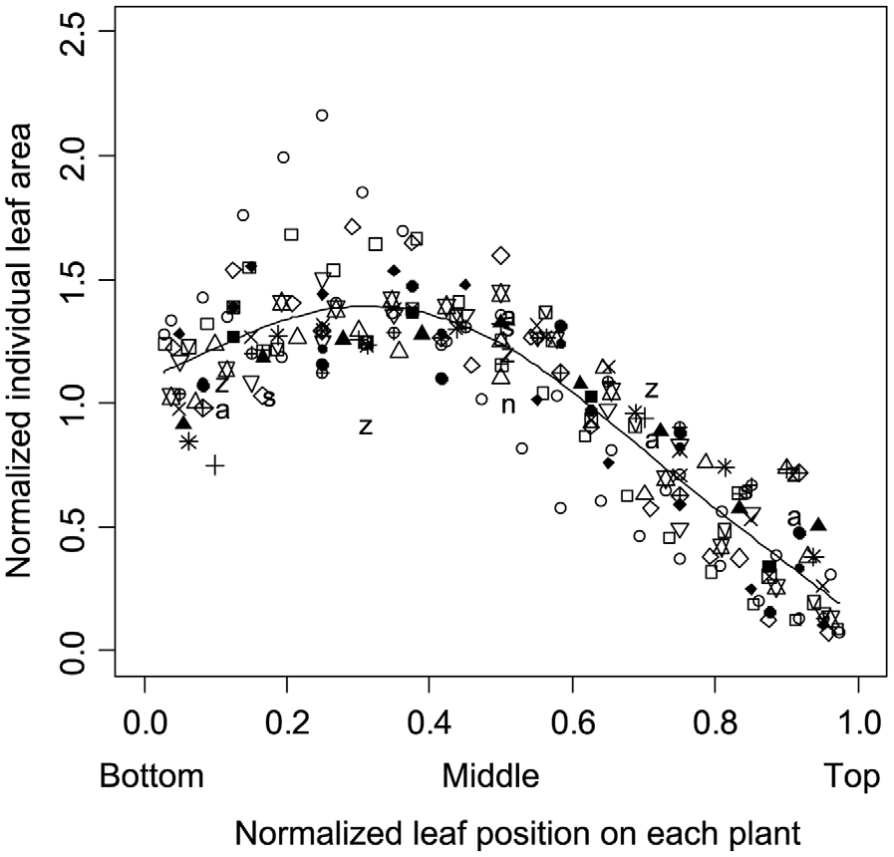
The normalized intra-plant leaf size distribution. Each symbol represents the relationship between the normalized position (relative number of leaves counted from the bottom to top of the stem, where 0 =  bottom, 0.5 =  middle and 1.0 =  top of the stem) and the normalized leaf area (area of an individual leaf divided by the averaged leaf area for the plant) of each leaf (*n* = 208). Each series shows an entire set of leaves for each plant (*n* = 29). The bold curve represents the fitted curve estimated by using the *gam* function in the *mgcv* package of R [29] (*r* ^2^ = 0.792).

**Figure 4:**
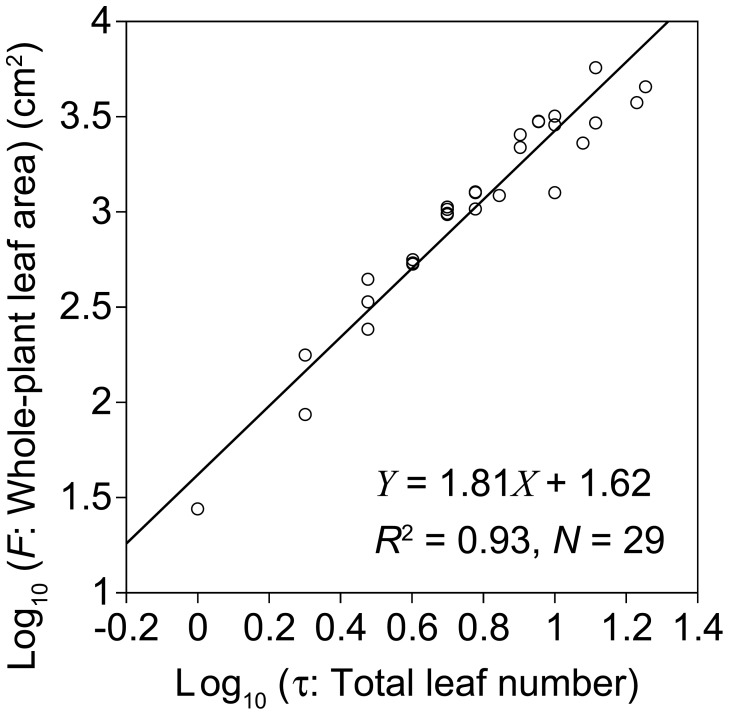
Log-log relationship between whole-plant leaf area and total leaf number. Each open circle represents one individual plant. The solid line shows the standardized major axis regression (*r* ^2^ = 0.93, *n* = 29).

#### Data analysis

Whole-plant leaf area (*F*) was calculated as the sum of all individual leaf areas on each plant. To describe the log-log relationship between whole-plant leaf area and total leaf number among different-sized plants, both standardized major axis (SMA) and ordinary least squares (OLS) regression lines were fitted by SMATR [28]. For each leaf, we defined a normalized leaf area and a normalized position. Normalized leaf area was defined as the area of a leaf divided by the mean individual leaf area of the plant to which the leaf was attached. This value was used to describe the intra-plant leaf size distribution on a normalized scale. The normalized position (*r*) of each leaf was defined as
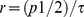
(1)where *p* is the absolute position of each leaf on each plant, and *τ* is the total leaf number of the plant to which the leaf was attached. The normalized position indicates the position of each leaf on a given plant (i.e. 0 =  the top, 0.5 =  the middle, and 1.0 =  the bottom of the plant). If we were to define the normalized position as *r* =  *p*/*τ*, it would produce a bias in that the single leaf on a single-leaf plant would have *r* = 1.0 (the lowest position). Instead, by Eq. 1, the single leaf on a single-leaf plant has *r* = 0.5 (the middle position). The rationale for this definition lies in the fact that a single leaf occupied each position from the top (*r* = 0) to the bottom (*r* = 1) on the plant, such that the mean value 0.5 should be applied. To examine the relationship between the normalized position of each leaf area and the normalized leaf area, we utilized an additive model [29]. The additive model was assessed with the *mgcv* package [29] using R version 2.14.2 [30].

## Results

Whole-plant leaf area varied by >200-fold (27.6–5690 cm^2^). In support of the dynamic scaling law, the relationship between normalized leaf position and normalized leaf area on each plant was similar among the different-sized plants (Fig. 3). On the basis of the additive model, the effect of normalized leaf position was highly significant, and normalized leaf area was well explained (*r* ^2^ = 0.792, *p*<0.001) (Fig. 3). In support of the power law, whole-plant leaf area (*F*) was a power function of total leaf number (*τ*) (Fig. 4). The SMA regression line (Log_10_ *F* (cm^2^)  =  *b* Log_10_ *τ* + *a*) had a slope and intercept of *b* = 1.81 (95% CI: 1.62–2.01) and *a* = 1.62 (1.46–1.78), respectively. Fitting the OLS regression line gave a similar result: *b* = 1.74 (1.55–1.93) and *a* = 1.67 (1.51–1.83), respectively (*r* ^2^ = 0.93, *p*<0.001). The slope (*b*) was larger than unity, indicating that mean individual leaf area (*F*/*τ*) increased with *τ* according to the power function: Log_10_ (*F*/*τ*)  =  (*b*–1) Log_10_ (*τ*) + *a*. Hence, both the size of the entire pattern (i.e., whole-plant size) and a unit pattern governing the entire pattern (i.e., single-leaf size) showed power-law dependence on developmental stage (*τ*).

## Discussion

Our results can further improve current metabolic theories of plant ecology. Specifically, current theories predict total leaf number (*τ*) as a power function of total plant mass (*M*) [i.e., *τ* = *k*_1_ *M^d^*], and predict whole-plant leaf area (*F*) by assuming that *F* is proportional to *τ* [16]. Our power-law results can be incorporated into these models as follows:
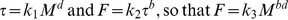
(2)


The dynamic scaling (Fig. 3) implies that the proportion of large (or small) leaves on a relative scale within a given plant is basically invariant among different-sized plants. In addition, within the largest *C. cordatum* plants, large leaves defined the canopy structure with small leaves filling the spaces between large leaves (Fig. 1). Accordingly, the overall canopy structure of large plants was similar to that of small plants (Fig. 1). These two phenomena, conservation of overall structure across developmental stages (i.e., dynamic scaling law), and the filling of spaces between large units with small units, are basic properties of fractal growth phenomena [31], and have been core assumptions of several models of plant allometry [4], [6], [7], [32]. Some models of plant development (e.g., the L-system [33]) can simulate fractal growth, in which idealized successive branching processes result in geometric similarity among different-sized plants across developmental stages. We observed the same properties for a plant species without an explicit, fractal-like hierarchical branching structure. The same basic template organizing plant form applied across developmental stages, facilitating conservation of efficiency of the light-capturing foliar array and mechanical stability. These results suggest the possibility of a form-balancing hypothesis, which predicts that scale invariance through development will be widespread in plants.

Studies of dynamic scaling have reported the power law as a function of physical time (e.g., seconds) under controlled laboratory conditions [17]–[19]. One difficulty in describing time-related power laws in biology derives from the fact that biological processes are affected by daily and annual cycles of temperature and light [34], [35], and by stochastic variance in external environments [35], [36]. To circumvent the problem of scaling plant growth to physical time, we used total leaf number (*τ*) as a measure of developmental stage; this is essentially the same as the plastochron index or plastochron age, which has been widely applied in studies of foliar development within a single growing season [11], [20]. By defining unit leaf production as a biologically meaningful time unit, we have filtered the effects of environmental variance, and have detected the applicability of the power law in this complex system.

There is an important limitation to the conclusions we have drawn from this study. We have obtained results for one species, from a single site. Further studies that examine a diversity of plant species and morphologies are needed to clarify the generality of these results.

## Supporting Information

Dataset S1 **All the data may be used with proper citation without contacting the authors.**
(CSV)
