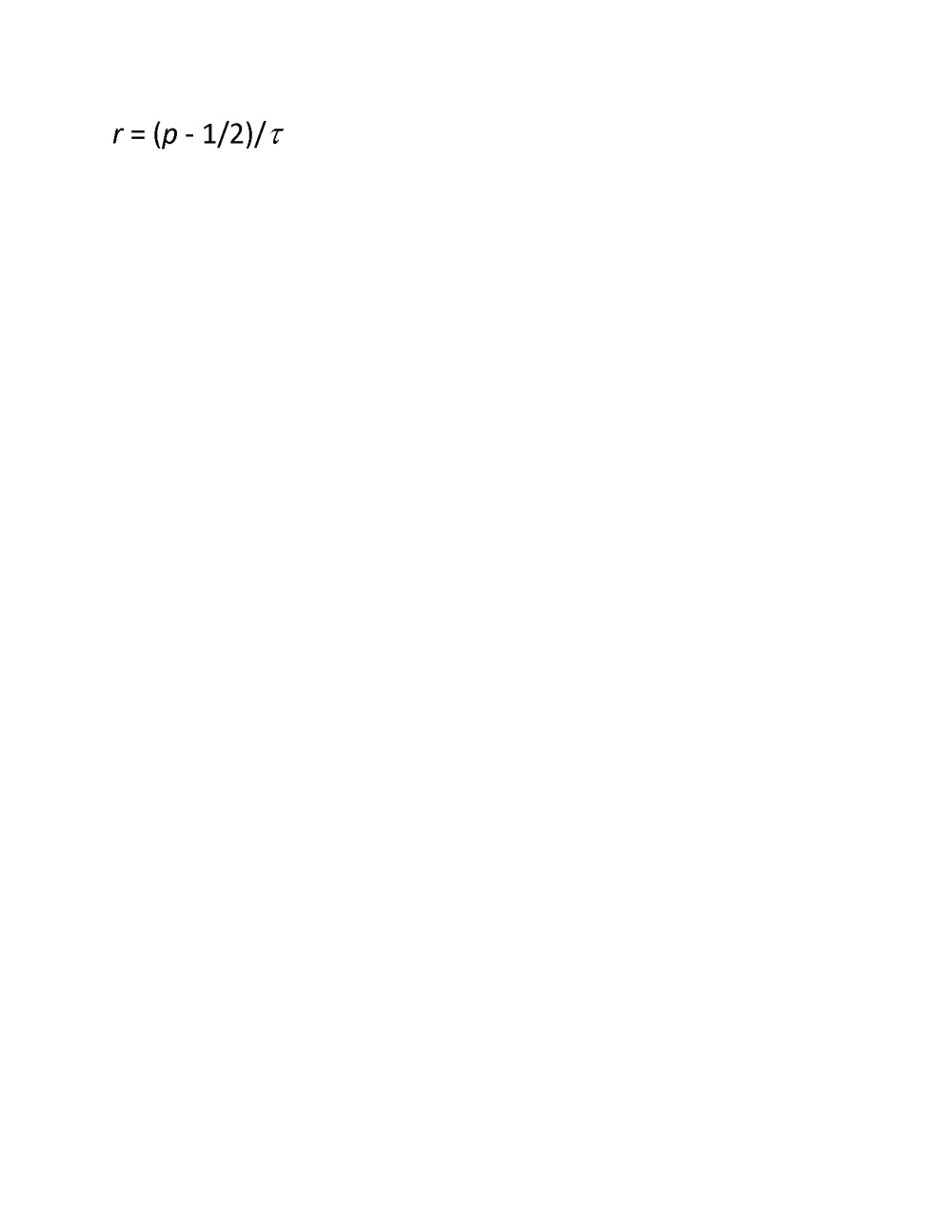# Correction: Dynamic Scaling in the Growth of a Non-Branching Plant, *Cardiocrinum cordatum*

**DOI:** 10.1371/annotation/adf4e7b0-d177-4d01-9419-1642f9a1318a

**Published:** 2012-11-14

**Authors:** Kohei Koyama, Yoshiki Hidaka, Masayuki Ushio

The correct Equation 1 can be viewed here: